# Gut bacteria of the cowpea beetle mediate its resistance to dichlorvos and susceptibility to *Lippia adoensis* essential oil

**DOI:** 10.1038/s41598-019-42843-1

**Published:** 2019-04-23

**Authors:** Mazarin Akami, Nicolas Yanou Njintang, Olajire A. Gbaye, Awawing A. Andongma, Muhammad Adnan Rashid, Chang-Ying Niu, Elias Nchiwan Nukenine

**Affiliations:** 1 College of Plant Science & Technology, Huazhong Agricultural University, Wuhan, 430070 China; 2 Department of Biological Sciences, Faculty of Science, University of Ngaoundere, P.O Box 454, Ngaoundere, Cameroon; 3 Department of Biology, Federal University of Technology, P.M.B. 704, Akure, Nigeria

**Keywords:** Symbiosis, Microbiome

## Abstract

Bacteria inhabiting the gut of insects provide many benefits to their hosts, such as aiding in food digestion, reproduction, and immunity, tissue homeostasis, adaptation to environment and resistance to pathogen and pesticides. The cowpea beetle, *Callosobruchus maculatus*, is a serious cosmopolitan pest of pulses. This beetle has lent itself as a guinea pig for several ecological studies. It harbors a consortium of bacterial communities in its gut, but the evidence for their role in its physiology is fragmentary. In this work, we hypothesized that gut microbiota mediates *C*. *maculatus* resistance to dichlorvos (DDVP or *O*,*O*-dimethyl *O*-2,2-dichlorovinylphosphate) and represent the target of *Lippia adoensis* (Gambian Tea Bush) essential oil (EO). Symbiotic and aposymbiotic beetles were exposed to artificial cowpea seeds earlier treated with DDVP or EO. Adult mortality and changes in gut bacterial community composition and abundance were examined at F_1_ and F_5_ generations. The susceptibility of experimental beetles to DDVP was significantly affected by their symbiotic status. The adult mortality decreased across generations in DDVP treatments, and remained significantly higher in aposymbiotic groups. In EO treatments, the mortality was consistent irrespective of symbiotic status and experimental generations. When compared to DDVP and the Control, EO treatments had significantly lower bacterial richness and diversity, as well as lower abundance of Proteobacteria, Firmicutes, and Bacteroidetes. These results support our hypothesis and describe the responses of gut microbial communities to pesticide treatments. This could be of interest for developing new management strategies of this pest.

## Introduction

Insects are associated with bacteria which colonize their body, cells and guts^1^ and contribute to their fitness in a variety of ways^2–4^. Bacteria play vital roles in host fitness and their interaction has greatly impacted the adaptation of host insects in their ecological niches^5,6^. For example, recent studies showed that gut endosymbionts contribute to host nutrition^7,8^, modulate host foraging behavior^9^ and enhance host resistance to entomophages^10^, and entomopathogens^11^.

Insect pest resistance is one of the major problems that characterize the use of synthetic insecticides. One of the many ways insects develop resistance to pesticides is through metabolic means such as detoxification. Generally, insects acquire resistance to pesticides through the expression (up-regulation or down-regulation) of detoxification enzymes^12–14^. The most commonly described are cytochrome P450 mono-oxygenase, glutathione-S-transferases and carboxylases^15,16^, which degrade xenobiotics into water soluble components easily excreted by the insects^17^.

Over the past decades, intestinal bacteria prompted interests for their prominent role in the process of detoxification which confer resistance in insects^7,18^. For example, Kikuchi *et al*.^19^ reported that bacteria of the genus *Burkholderia* impart protection against organophosphorus pesticides in stinkbugs. Intestinal bacteria from the fifth instars larvae of *Spodoptera frugiperda* were shown to degrade synthetic insecticides lambda-cyhalothrin, deltamethrin, chlorpyrifos ethyl, spinosad and lufenuron^7^.

The cowpea beetle, *Callosobruchus maculatus* (F.) (Coleoptera: Chrysomelidae), is the most destructive pest of stored cowpea worldwide^20^. This pest infests cowpea from the field before harvest and causes substantial damages to the stored seeds, owing to its short biological cycle (25–28 days) and higher fecundity rates^21^. The adults are not harmful as they do not require any food or water along their lifespan (~2 weeks)^22,23^, but mate multiple times to produce eggs and sustain their progeny^24,25^. The adults are ready for mating within 24–36 hours post emergence, and search for oviposition substrate (seeds)^22^. The larvae bore into the seeds’ endosperm, undergo a series of molts^26^ and cause considerable quantitative and qualitative losses in the storage. Moreover, this pest is reported to harbor rich and diverse bacterial communities in their guts^27,28^ which can play a role in host resistance and adaptation to pesticides^29^. However, studies on how gut bacterial communities structure and composition are affected when *C*. *maculatus* is exposed to pesticides across multiple generations are still fragmentary.

**Figure 1:**
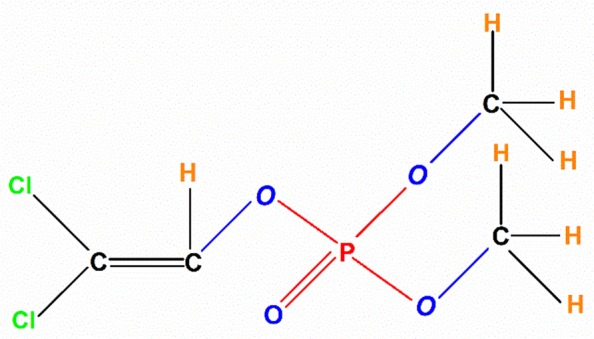
Chemical structure of dichlorvos (*O,O*-dimethyl,*O*-2,2-dichlorovinylhosphate).

Dichlorvos (*O*,*O*-dimethyl *O*-2,2-dichlorovinylphosphate or DDVP) (Fig. 1) is one of the most popular organophosphate (OP) insecticides widely used to control *C*. *maculatus* in developing nations, such as Cameroon and Nigeria^20,30^. However, the repeated use of this OP has led to an increase in its level in several ecosystems and development of resistance in *C*. *maculatus* populations^31^.

Essential oils (EOs) are secondary metabolites produced by aromatic plants to protect themselves from herbivorous insects^32,33^. One of such aromatic plant is *Lippia adoensis* Hochst, commonly known as “Gambian Tea Bush”, “Bush Tea”, “Healer Herb” or “Butter Herb”^34,35^. It is known locally as “*Ligi or Gossolderi*” (in Fulfulde language) or “Fever Tea” in northern Cameroon. *Lippia adoensis* EO is reported previously to possess a variety of chemical constituents, mostly monoterpenes (supplementary), which confer on them a broad spectrum of insecticidal activities^36^. EOs are less toxic to the environment (biodegradable), humans and non-target organisms and their chemical constituents have multiple modes of actions on insects^37^. They are also reported to target the gut microbiome and suppress their contribution in the establishment of resistance in insects^38,39^.

In this study, we examine the hypothesis that gut microbial communities could mediate and/or sustain *C*. *maculatus* resistance to DDVP. In contrast, *L*. *adoensis* EO could target gut bacterial communities (including those that are resistant to DDVP) by disrupting their activities in order to enhance the susceptibility of the beetle to the toxin. We produced aposymbiotic beetles from the larval stage (as adult does not feed) by inoculating two antibiotics (Ciprofloxacin and Gentamycin) in artificial cowpea seeds on which normal eggs were allowed to develop. Likewise, the experimental seeds were produced by applying DDVP or EO on artificial cowpea seeds, to which, symbiotic and aposymbiotic beetles were exposed across multiple generations. The effects of this long term exposure to both pesticides on adult mortality and on the structure, composition and diversity of gut microbiota were assessed at first and fifth generation.

## Results

### Adult mortality

**Figure 2:**
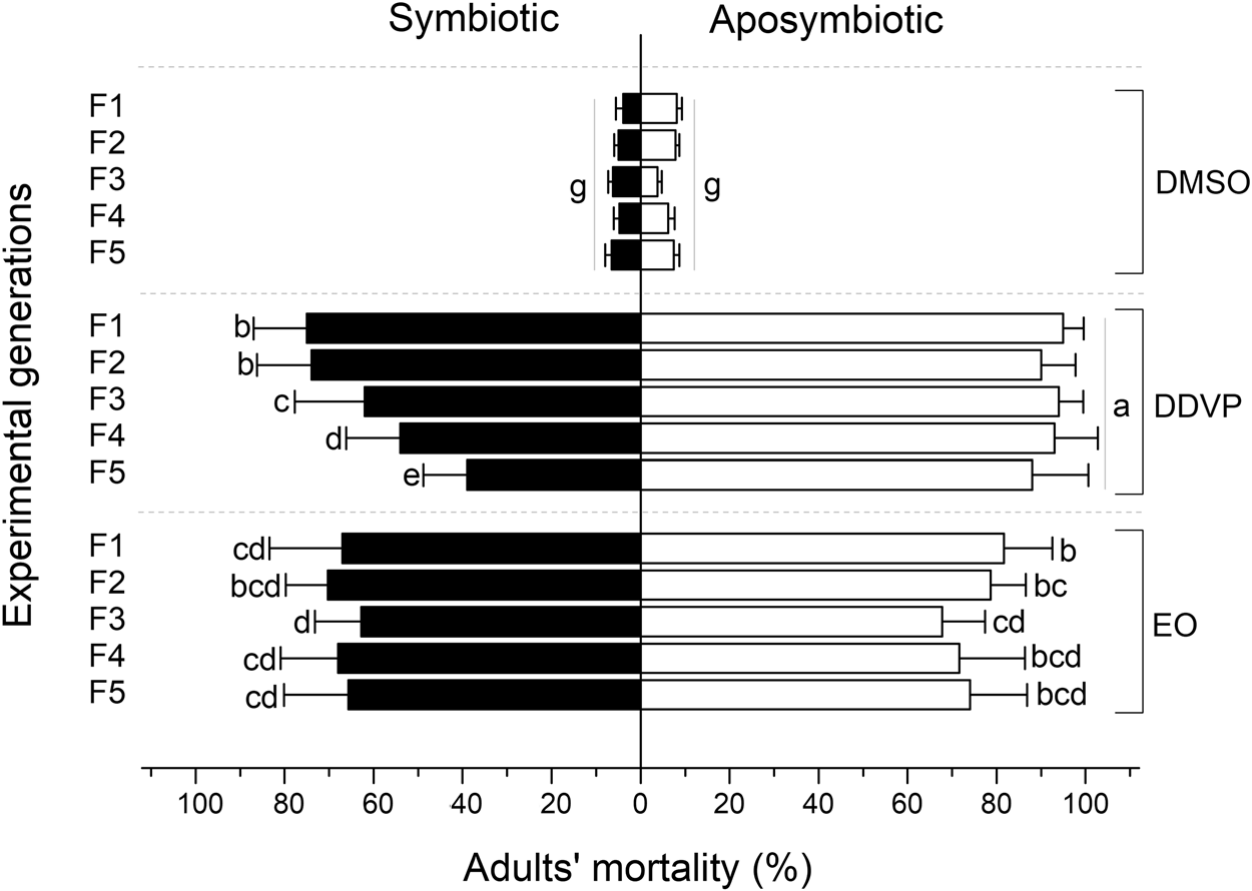
Variation in the susceptibility of symbiotic and aposymbiotic beetles over five generations. Means with different letters between generations of each pesticide treatment are significantly different after comparison with parametric New Duncan’s Multiple Range Test at p = 0.05.

The susceptibility of experimental beetles was significantly affected by symbiotic status (Ordinary Least Squares Regression Model, F = 95.952; df = 1 r^2^ = 0.988; t = 3.936; P < 0.0001), pesticides used (F = 191.036; df = 2; r^2^ = 0.969; t = 4.598; P < 0.0001) and by experimental generations (F = 8.531; df = 4; r^2^ = 0.895; t = 2.751; P < 0.0001). However, the susceptibility of aposymbiotic beetles treated with DDVP did not vary significantly across generations (F = 94.95; df = 2; r^2^ = 0.673; t = 3.481; P = 0.148) (Fig. 2). The symbiotic status and experimental generations did not affect the susceptibility of beetles treated with DMSO only (control) (F = 28.894; df = 1, 4; r^2^ = 0.776; t = 2.027; P = 0.282) as very low and similar mortality was recorded between the symbiotic and aposymbiotic beetles across generations (Fig. 2).

Overall, aposymbiotic beetles were the most susceptible populations to DDVP and *L*. *adoensis* EO, compared to symbiotic ones (F = 350.245; df = 1; P < 0.0001 and F = 19.245; df = 1; P < 0.0001) (Fig. 2). The mortality of symbiotic beetles significantly decreased from the third generation in DDVP treatments (ANOVA, F = 20.382; df = 4; P < 0.0001), while it remained higher but consistent across generations in aposymbiotic beetles (F = 65.34; df = 4; P = 0.667) (Fig. 2). The successive generations did not significantly affect the susceptibility of *C*. *maculatus* to *L*. *adoensis* EO (F = 13.302; df = 4; P = 0.5058 and F = 13.302; df = 4; P = 0.274 for symbiotic and aposymbiotic beetles, respectively) (Fig. 2).

### Diversity of operational taxonomic units (OTUs)

A total of 63,709 raw reads were generated from all treatments (Control, DDVP and EO) at F_1_ and F_5_ generations (35,069 and 28,640 reads, respectively). After quality trimming, filtering and downstream analyses, 55,620 high quality sequences were obtained (31,328 and 24,292 from F_1_ and F_5_, respectively) and generated on average 194 ± 69 OTUs (205 ± 37 and 183 ± 100 OTUs from F_1_ and F5, respectively). Experimental generations did not significantly affect the OTUs composition of the Control and DDVP treated samples (Ordinary Least Squares Regression Model, F = 0.137; r^2^ = 0.86; t = 0.688; df = 1; P = 0.73 and F = 54.16; r^2^ = 0.86; t = 0.688; df = 1; P = 0.26, respectively) (Table 1). However, the number of OTUs from EO treated samples varied significantly across generations (Ordinary Least Squares Regression Model, F = 27.021; r^2^ = 0.939; t = 1.73; df = 1; P = 0.007), and was significantly reduced compared to the Control and DDVP treatments (ANOVA, F = 7.078; df = 2; P < 0.001 and F = 7.078; df = 2; P < 0.0001, respectively) (Table 1). There was a non-significant increase of OTUs number at F_5_ in DDVP treatments compared to their amount at F_1_ (ANOVA, F = 7.82; df = 1; P = 0.056) (Table 1).

**Table 1:** Richness and diversity parameters of gut microbiome from *C*. *maculatus* as affected by DDVP and EO.

Generations	F_1_			F_5_		
Parameters	Control	DDVP	EO	Control	DDVP	EO
Coverage (%)	77.08 ± 2.9^b^	71.96 ± 1.56^b^^c^	87.38 ± 2.19^a^	68.4 ± 1.32^c^	72.08 ± 2.24^bc^	89.42 ± 1.54^a^
OTUs^1^	74 ± 4^a^	77 ± 5^a^	54 ± 6^b^	76 ± 4^a^	84 ± 5^a^	22 ± 3^c^
Chao1	7.39 ± 1^ab^	7.47 ± 0.04^a^	5.93 ± 0.15^c^	6.87 ± 0.18^b^	7.18 ± 0.1^ab^	4.47 ± 0.3^d^
Shannon^2^	4.78 ± 0.13^a^	4.98 ± 0.1^a^	3.58 ± 0.3^b^	4.61 ± 0.14^a^	4.56 ± 0.15^a^	2.47 ± 0.11^c^
Simpson^2^	0.92 ± 0.02^a^	0.94 ± 0.01^a^	0.67 ± 0.03^c^	0.82 ± 0.03^b^	0.76 ± 0.04^d^	0.57 ± 0.03^d^
IL (kb)^3^	1.3–1.5	1.3–1.5	1.3–1.5	1.3–1.5	1.3–1.5	1.3–1.5
Cutoffs	0.0001	0.0001	0.0001	0.0001	0.0001	0.0001

Different letters (^a, b, c, d^) within rows are statistically different after parametric New Duncan’s Multiple Range Test at P = 0.05. ^1^The operational taxonomic units (OTUs) were defined with 3% dissimilarity level; ^2^The richness estimator (Chao1) and diversity indices (Shannon and Simpson) were calculated according to Good’s method^72^ and the mothur program, respectively; ^3^Isoform length (kb). Each datum represents the Mean ± SE of 3 replicates.

**Figure 3:**
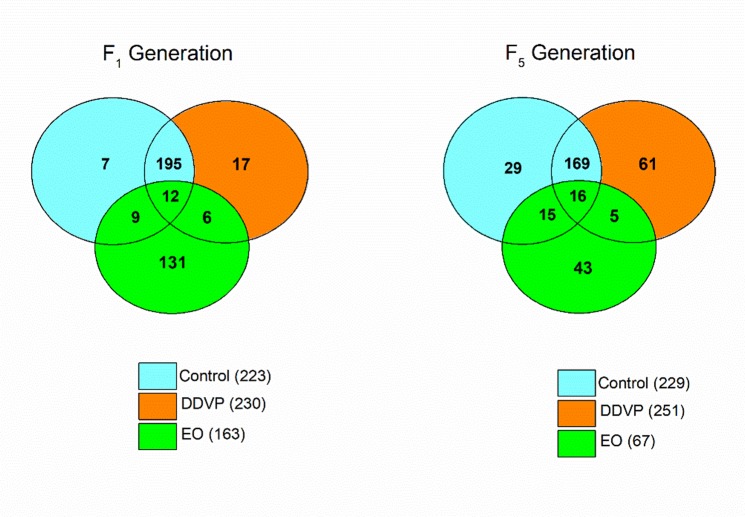
Venn diagram showing the distribution of unique and shared OTUs within and between samples.

Overall, of the 615 OTUs and 547 OTUs detected from all samples at F_1_ and F_5_, respectively, only 12 and 16 core OTUs were shared among the three samples at F_1_ and F_5_, respectively (Fig. 3). The Control and DDVP treated samples shared the majority of OTUs detected at F_1_ and F_5_ generations (with 195 OTUs and 169 OTUs, respectively), but the EO treated samples recorded the highest number of unique OTUs at F_1_ generation (131 OTUs) (Fig. 3).

### Bacterial richness and diversity

The non-parametric richness indexes (Shannon and Simpson) evaluated at 97% similarity, showed similar comparative trends in the prediction of the number of OTUs from DDVP and Control samples. The Shannon diversity index provides not only species richness (i.e. the number of species present) but how the abundance of each species is distributed (the evenness of the species) among all the species in the community. Here, the bacterial diversity were similarly higher in the Control and DDVP samples at both tested generations (Control: Shannon = 4.78 ± 0.13 and Shannon = 4.98 ± 0.1; DDVP: Shannon = 4.61 ± 0.14 and Shannon = 4.56 ± 0.15, at F_1_ and F_5_, respectively), but significantly lower in EO samples (Shannon = 3.58 ± 0.3 and Shannon = 2.47 ± 0.11, at F_1_ and F_5_, respectively) (F = 1699.178; df = 1; P = 0.032) (Table 1).

The Multi Response Permutation Procedure (MRPP) analysis based on Bray-Curtis distances revealed no significant difference between the gut microbiotas of Control and DDVP treated beetles (A = 0.09208; P = 0.558). However, the microbiome of EO treated beetles was found to be significantly different from the gut microbiomes of the Control and DDVP treated beetles (Bray-Curtis distance statistics, A = 0.009871; P = 0.0089 & A = 0.0002764; P < 0.0201). Similar trends were observed after the analysis of similarity (ANOSIM) performed on the various treatment groups. There was no significant difference in the structure, composition and abundance of the Control and DDVP treated beetles (R = 0.7518, P = 0.201), which were both different from the EO treated beetles (EO- Control: R = 0.798; P = 0.0014 & EO-DDVP: R = 0.9602; P < 0.001).

### Rarefactions

**Figure 4:**
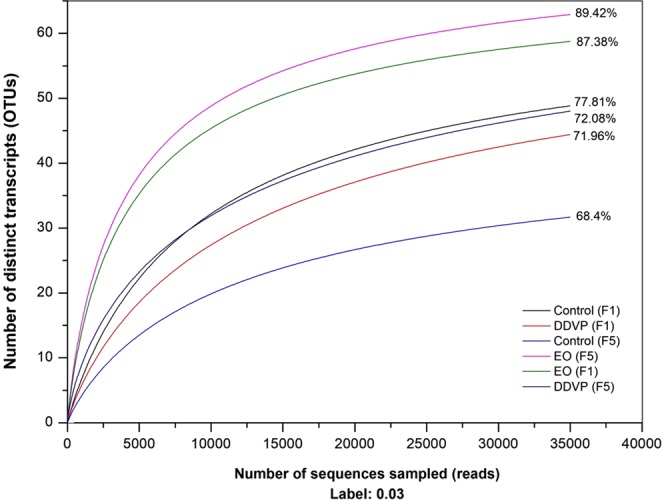
Multiple samples rarefaction curves based on 16S rRNA genes pyrosequencing. This was used to evaluate the completeness of the sequencing effort in describing the diversity of guts associated bacteria of *C*. *maculatus*. The OTUs were defined by 3% distances. Percentage values represent coverage indices which are given from a Chao1 analysis.

The rarefaction is a computational analysis of species accumulation based on the repeated re-sampling of all clusters. The curve is therefore the representation of statistical expectation for the observed accumulation curves, enabling the comparison of the statistically expected species richness of each community at the same sampling effort or abundance. The flattening of the rarefaction curves recorded here indicates that the sampling was of adequate depth and that additional sampling would produce few additional operational taxonomic units (OTUs) (Fig. 4).

### Bacterial diversity analysis

#### At Phylum level

**Figure 5:**
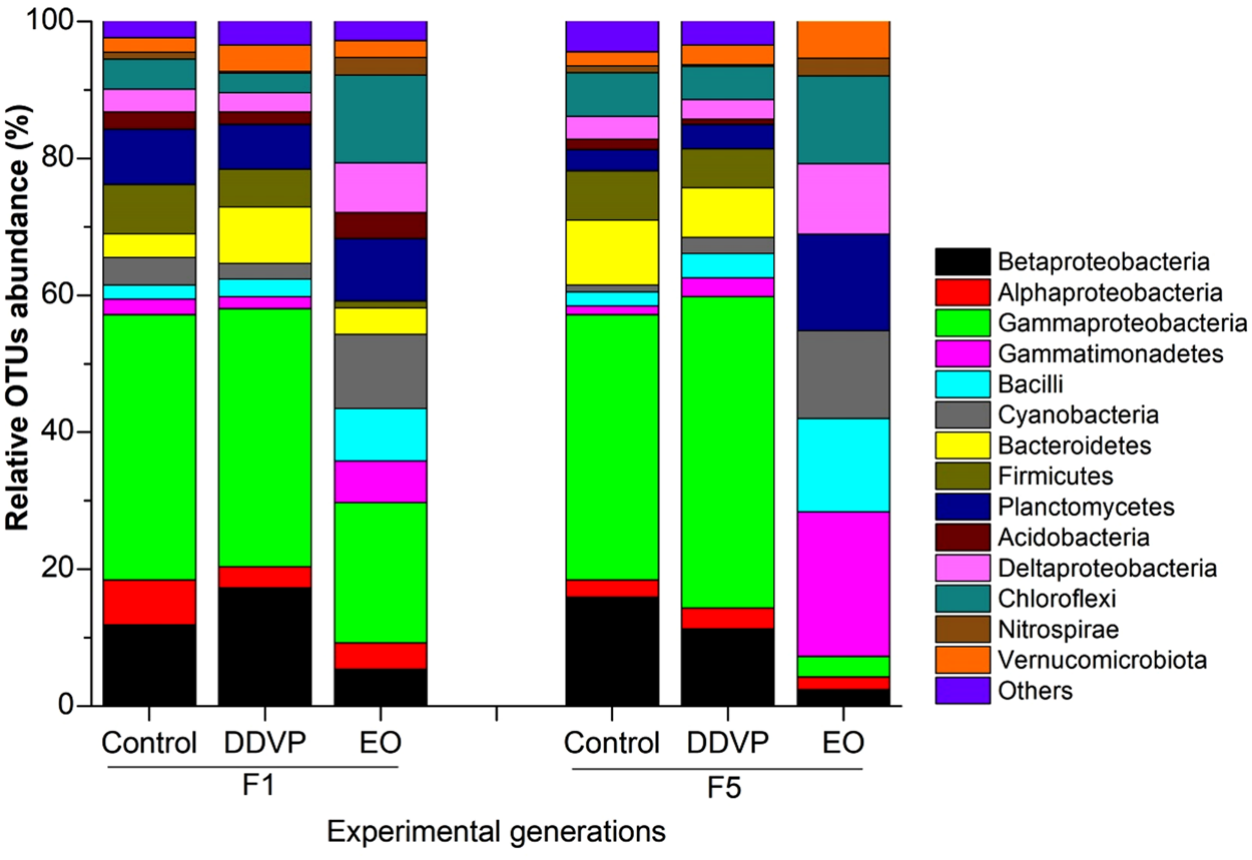
Relative abundance of the dominant bacterial phyla in all samples revealed by 454-pyrosequencing. The relative number of OTUs was defined as the percentage of the species sequences in total successfully classifiable sequences in the samples using SILVA databank. Data was obtained at an average threshold of 97%. Phylogenetic groups accounting for less than 1% of all classified sequences are summarized as “Others”.

Proteobacteria (gamma: 35.60% and beta: 13.26%), Bacteroidetes (7.89%) and Firmicutes (6.09%) were the most dominant bacterial phyla equally represented at F_1_ and F_5_ generations in DDVP and Control samples, although their proportions were slightly different (Fig. 5). The proportions of gammaproteobacteria, betaproteobacteria and Firmicutes were significantly reduced at F_1_ and F_5_ in EO samples in comparison with the Control and DDVP. Bacteroidetes, Firmicutes and Acidobacteria present at F1 in EO samples were completely missing at F_5_. Gammatimonadetes, Bacilli, Cyanobacteria, Chloroflexi and Deltaproteobacteria detected in low abundance (≤2%) in the Control and DDVP (F_1_ and F_5_) samples, proliferated significantly in EO samples at F_1_ and F_5_(Fig. 5). Overall, the gut bacterial communities in *C*. *maculatus* were dominated by Proteobacteria (γ, α & β) (53.67 ± 11.36%), Bacteroidetes (7.87 ± 3.61%) and Firmicutes (6.09 ± 2.04%) (Fig. 5). Other bacterial phyla consisted of Planctomycetes, Chloroflexi, Verrucomicrobiota and unclassified taxa (others) (Fig. 5).

#### At Family and genus levels

**Figure 6:**
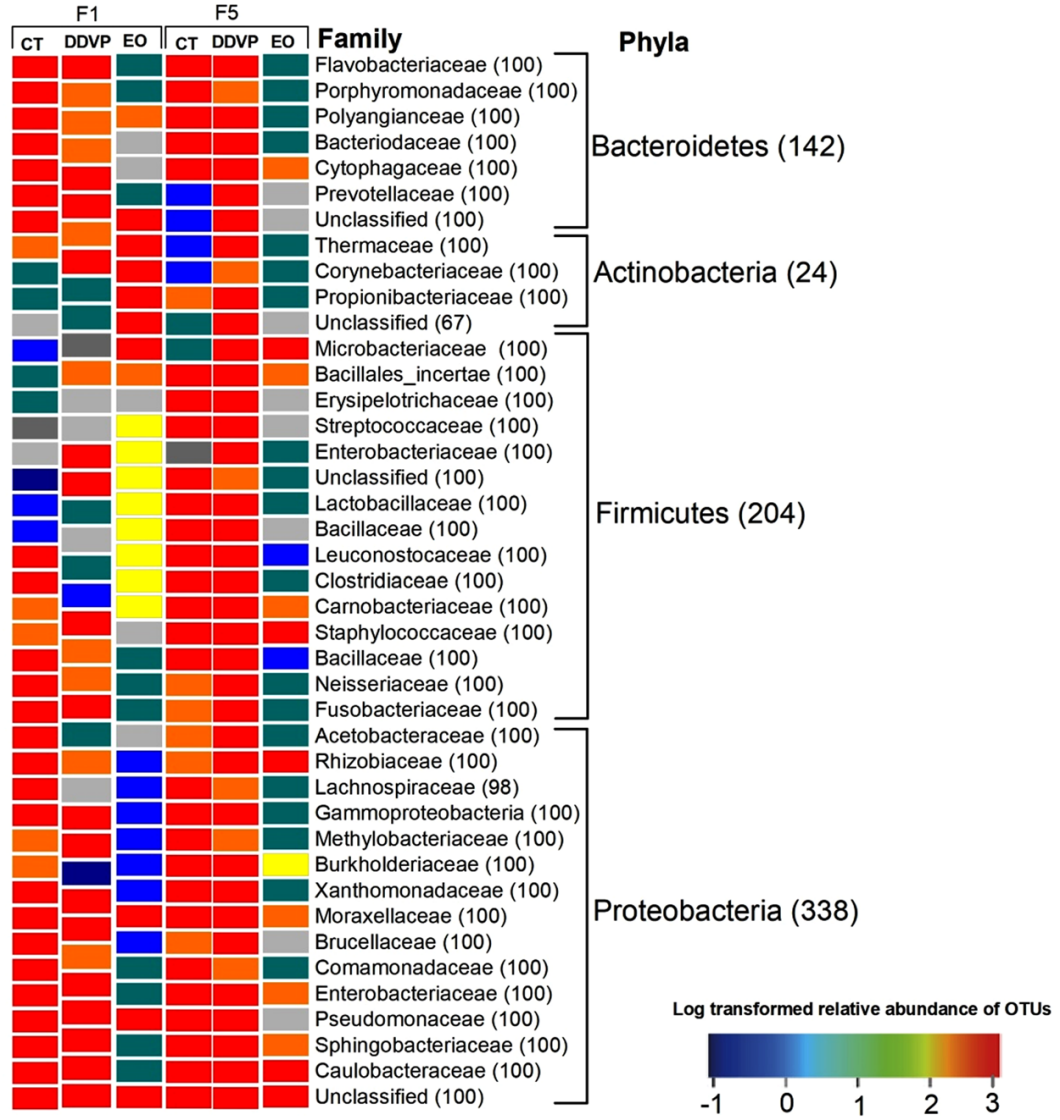
Gut bacterial community composition of *Callosobruchus maculatus* as affected by DDVP and EO. The heat map represents the log-transformed relative abundance of each bacterial family from each sample and the blue colors represent the absence of OTUs. Only high quality trimmed sequences were used to generate the heat map. The non-abundant OTUs (<1%) are not represented in this chart. The distance of log-transformed clusters from the three samples and bacterial families are indicated in the right side of the figure. The numbers between parentheses in front of each taxon represent their relative abundance. DDVP: O,O-dimethyl,O-2,2-dichlorovinylphosphate; EO: essential oil; CT: control.

The OTUs assigned to Proteobacteria matched to the classes alpha, beta, delta and gammaproteobacteria and to eleven bacterial families (Comamonadaceae, Enterococcaceae, Enterobacteriaceae, Methylobacteriaceae, Moraxellaceae, Acetobacteraceae, Morganellaceae, Pseudomonadaceae, Sphingomonadaceae, Rhizobiaceae, and Polyangiaceae); four families from Firmicutes (Staphylococcaceae, Carnobacteriaceae, Lactobacillaceae, and Streptococcaceae); four families from Bacteroidetes (Bacteroidaceae, Porphyromonadaceae, Sphingobacteriaceae, and Flavobacteriaceae) and finally three families from Actinobacteria (Micrococcaceae, Brevibacteriaceae and Propionibacteriaceae) (Fig. 6). Other bacterial families were represented by only a few bacterial sequences. The percentage of unclassified sequences was not taken into consideration during the calculation of their abundance. Overall, 51.69% of bacterial genera were identified from the different samples, representing a total of 42 genera. Most of the dominant genera were from Proteobacteria: these are *Acinetobacter* (4.73%), *Pseudomonas* (2.03%), *Citrobacter* (2.03%), *Klebsiella* (1.69%), *Orbus* (1.69%) and *Comamonas* (1.03%). The Firmicutes were predominantly represented by *Lactobacillus* (3.04%), *Lactococcus* (3.04%), *Staphylococcus* (2.03%) and *Exiguobacterim* (1.35%). *Dysgonomonas* (9.80%) is the most dominant genus from Bacteroidetes phyla. Burkholderiales represent 10.93% of the total bacterial order detected although no genus was identified.

#### Bacterial structure distribution

**Figure 7:**
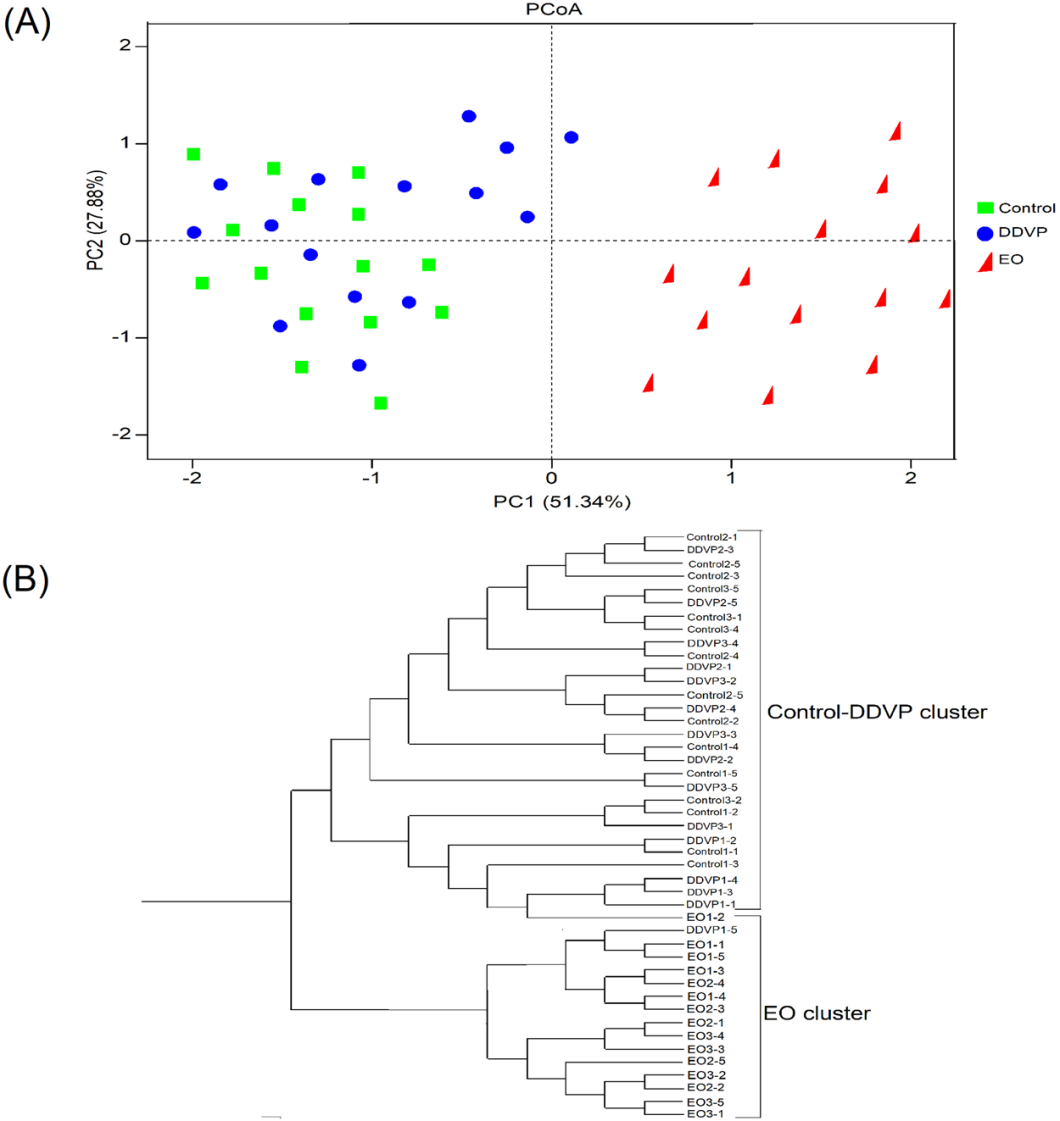
Multivariate Principal Coordinates Analysis (PCoA) plots showing the distance between *C*. *maculatus* gut samples (**A**). Each color point represents individuals from each treatment group. The cladogram represents the clustering of the different samples based on the composition of their microbiotas (**B**). The analysis was created by using an average linkage from the weighted UniFrac distance matrices. Each node supported by a *Jackknife* analysis with greater than 100% accuracy. This analysis was based on the relative abundance of bacterial phyla and proteobacterial classes. Figures before treatment groups represents the various biological replicates and sample ID (for example, Control1-1 means first biological replicate and the first individual from the control group).

Bacterial community structure did not vary significantly across generations in Control and DDVP treated samples (F = 581.677; df = 1; P = 0.158 and F = 581.677; df = 1; P = 0.058, respectively) (Fig. 7A), but vary significantly in EO treated samples at F_1_ and F_5_ (F = 1699.178; df = 4; P < 0.001 and F = 1699.178; df = 4; P < 0.0001, respectively) (Fig. 7A). A cladogram representing the clustering of the different samples based on the composition of their microbiotas show that the Control and DDVP groups are closely related but both are distantly related with the EO treated beetles (Fig. 7B).

## Discussion

Several insects have developed extraordinary capacity to survive exposure to xenobiotics normally designed to manage them^40^. Furthermore, insects harbor a wide variety of gut symbionts^2,41,42^, which play pivotal roles in their adaptation to the environment following exposure to pesticides^19,43^. In an attempt to reduce the incidence of insecticide resistance in *C*. *maculatus*, coupled with the necessity of developing an alternative control strategy, we tested the hypothesis that gut microbiota of *C*. *maculatus* are involved in its resistance to dichlorvos and *L*. *adoensis* EO could break this resistance and enhance the susceptibility of the beetle across generations.

On one hand, the results showed that the adult mortality in both pesticides was significantly higher in aposymbiotic beetles compared to the symbiotic ones and the control groups. On the other hand, the susceptibility of symbiotic *C*. *maculatus* declined from the third generation in DDVP treatments whereas in EO treatments, it remained consistently higher across all tested generations (Fig. 2). These findings suggest that symbiotic beetles have developed the ability to tolerate DDVP molecules, presumably sustained by their gut bacteria, as their population crash in aposymbiotic beetles resulted in significant increase of their susceptibility compared to the symbiotic ones and remained consistent across generations.

The mortality of EO treated beetles (symbiotic and aposymbiotic) was not significantly affected across generations, contrary to DDVP where the susceptibility of symbiotic beetles dropped irrevocably from F_3_. This suggests that, the symbiotic beetles started developing resistance to DDVP from F_3_ generations and the sustainability or heritability of the resistance trait in the following generations depended on the symbiotic status and pesticides used. After F_3_, *L*. *adoensis* EO succeeded to reduce resistance in the following generations, thereby causing failures in the beetle’s adaptation attempts or defense mechanism mediated bacterially. Previously, gut microbial symbionts were shown to be implicated in insect-plant interactions by increasing the adaptations of insects to plant toxins^17,44^. The results from this study showed an opposite trend whereby allelochemicals of *L*. *adoensis* EO sustained the susceptibility of *C*. *maculatus* potentially by reducing the most dominant populations of gut bacteria.

The 454-pyrosequencing analysis of gut microbiome provided empirical evidence on bacterial phyla potentially involved in the interaction with DDVP and EO. Proteobacteria, Bacteroidetes and Firmicutes were abundantly detected in the Control and DDVP treated samples at F_1_ and F_5_ generations (Fig. 5). However, their proportions in EO treated beetles were significantly reduced across generations and these correlated with the increase in adult mortality. Possibly, these bacterial phyla could be associated to DDVP-biodegradation in *C*. *maculatus*, allowing the host to survive exposure to DDVP across generations by increasing the host tolerance to it. Moreover, the presence of the genera *Acinetobacter*, *Citrobacter*, *Pseudomonas* and Burkholderiales (although unclassified) genera in *C*. *maculatus* gut could be associated to their ability to develop resistance to DDVP components. Similarly, recent studies have shown that bacterial species from Proteobacteria phyla of the genera *Citrobacter*, *Burkholderia* and *Pseudomonas* were implicated in biodegradations of trichlorphon and fenitrothion (both OP pesticides) by *B*. *dorsalis* and *Bemissia tabacci* as well as in the environment^29,45,46^.

Conversely, when *C*. *maculatus* was treated with *L*. *adoensis* EO, the populations of Proteobacteria, Bacteroidetes and Firmicutes (the most dominant bacterial phyla in untreated beetles) were significantly reduced and the vulnerability of treated beetles increased and remained consistent across generations. This could be an indication that Proteobacteria, Bacteroidetes and Firmicutes may represent to some extent, the primary targets of chemical constituents of *L*. *adoensis* EO. Recent studies on insect gut microbiology have shown that Proteobacteria and Firmicutes were the most dominant bacterial phyla in the midgut of Lepidopterans, including *Lymantria dispar*, *Helicoverpa armigera*, and *Bombyx mori*^47–49^. At the family level, Enterococcaceae was the most abundantly represented in the gut of *C*. *maculatus*, followed by Enterobacteriaceae (Fig. 6) as earlier discovered in three life stages of *Bactrocera dorsalis*^50^. This could be an indication that Enterococcaceae and Enterobacteriaceae might play important roles in ecological adaptations and survival of *C*. *maculatus*.

The use of antibiotics to suppress the gut microbial communities did not affect the survival of aposymbiotic beetles as the very little mortality recorded was similar to that of the symbiotic ones. This suggests that the mortality recorded was not caused by the use of antibiotics (in aposymbiotic beetles), but by the pesticide treatments (EO and DDVP). However, the suppression of gut bacteria by antibiotics resulted in higher adult mortality rates compared to the symbiotic ones. This suggests that, without its intestinal bacterial, *C*. *maculatus* could not successfully survive exposure to DDVP. The high number of chemical constituents detected from *L*. *adoensis* EO^26^ may come into play toward maintaining the consistent susceptibility of the beetles across generations by targeting the most abundant bacterial communities (Fig. 5). Many previous works demonstrated the interactions between gut bacteria and phytochemicals^51–54^, but the specific conserved functions of gut bacteria in the cowpea beetles need further investigations of the whole transcriptomes of the insect. At the very least, our results give evidence that gut bacterial communities in *C*. *maculatus* might mediate DDVP degradation making them resistant to it. These bacteria might also be the target of plant toxins from EO making *C*. *maculatus* more susceptible to phytochemicals. We therefore believe that plant allelochemicals may have evolved antibiotic functions to disrupt gut bacterial communities in *C*. *maculatus*.

On the basis of previous transcriptomic and microarray analyses carried out on *C*. *maculatus* gut^55,56^, the future works will focus on evaluating the expression profiles of detoxification genes to understand the extent of gut microbiome involvement in molecular resistance or susceptibility in this cowpea pest.

## Conclusion

The development of resistance in pest insects is a major challenge to sustainable agriculture and pest management. The present study demonstrated that the Control and DDVP treated *C*. *maculatus* shares similar core bacteria, dominated by Proteobacteria, Bacteroidetes and Firmicutes. These bacterial phyla may allow the adults *C*. *maculatus* to survive on DDVP treated grains, thereby making it inappropriate to control the beetle populations in the field. The results also revealed that *L*. *adoensis* EO could represent an alternative pesticide to DDVP through its capacity to uphold the vulnerability of the beetles across generations, hence maintaining their populations below economic thresholds. From an ecological perspective, the findings herein broaden our understanding of the implications of gut bacteria in the adaptation mechanisms of *C*. *maculatus* to pesticides. This could help to develop new tools to aid the implementation of eco-friendly control strategies and reduce the environmental impact of synthetic chemicals.

## Methods

### Insects rearing and maintenance

Twenty kilograms of dried cowpea seeds (*Vigna unguiculata*) (black eye beans) were collected from Lara locality in Northern Cameroon (latitude 10°10′00.0″N and longitude 14°31′00.0″E). The seeds were cleaned and disinfested by storing them at −20 °C for 10 days and thereafter left undisturbed under laboratory conditions (27 ± 3 °C, 55 ± 5% relative humidity and 10: 14 Light: Dark photoperiod) for 5 days for acclimatization and to avoid moldiness^20^. To minimize variations in the insect populations, adult *C*. *maculatus* that emerged from infested field collected seeds were reared on disinfested cowpea seeds for about eight generations until required for bioassays.

### *Lippia adoensis* essential oil (EO) and dichlorvos (DDVP)

Fifty grams of fresh *L*. *adoensis* leaves were harvested at the vicinity of the University of Ngaoundere, Cameroon (latitude 7°19′39″N and longitude 13°35′04″E), shade-dried for one week and ground. The essential oil was extracted by hydro-distillation using a modified Clevenger-type apparatus (XWD-C-1000, Shanghai XinWangDe Laboratory Equipment Co., China) for 6 h and the oil was extracted with *n*-hexane. The recovered crude essential oil (EO) was stored in an airtight glass container and kept at 4 °C^20^. The chemical constituents of *L*. *adoensis* EO were determined and quantified by Gas chromatography/mass spectrometry as previously described^26^. Dichlorvos or DDVP (*O*,*O*-dimethyl,*O*-2,2-dichlorovinylphosphate) 100 EC 10^3^ g/L (PESTANAL®, Analytical Standard, C4H7Cl2O4P) was obtained from Sigma-Aldrich (China) (ID: 45441, molecular weight: 220.98; CAS number: 62-73-7) at 100% purity.

The EO and DDVP were separately dissolved at equal proportions (1:1) in dimethylsulfoxide or DMSO (solvent) prior to treatments.

### Artificial cowpea grains

Five kilograms of disinfested cowpea seeds were soaked in distilled water for 30 minutes to allow the removal of testae. Decorticated seeds were air-dried overnight under room temperature and ground into fine flour. The resulting flour was used to prepare two types of artificial seeds resulting flour (see details in Supplementary 1(3)). The normal seeds (flour without any antibiotics), and the antibiotic groups (flour mixed with 10 µg of Ciprofloxacin per gram of flour and 4 µg of Gentamycin per gram of flour at the preparation)^55^. Mineral elements (methyl-p-hydroxybenzoate, choline chlorite, L-ascorbic acid and sodium benzoate) were added among the ingredients to compensate those removed during seed decortication and to boost the females’ fecundity.

The concentrations of antibiotics represent their minimum inhibitory concentration (MIC) and the two antibiotics were selected for their potencies to clear the gut microbiota of *C*. *maculatus* based on the colony forming units (CFU) of gut homogenates (Supplementary 1). In each treatment group, the artificial cowpea seeds were left undisturbed for 24 h and equilibrated with atmospheric moisture, prior to coating with 10% gelatin^55^. The preparation procedures were carried out under aseptic conditions. The artificial cowpea seeds were air-dried under a laminar flow hood and packaged in plastic bag until use for bioassays.

### Production of symbiotic and aposymbiotic beetles

10 mated *C*. *maculatus* couples were introduced in two separate glass jars (500 mL size), respectively, each containing 50 g of normal artificial seeds and antibiotic treated seeds, respectively. The females were allowed to lay eggs on the seeds in which the larvae were allowed to develop till adult emergence. The beetles emerging from the normal seeds are symbiotic (containing intact gut microbiome) and those emerging from antibiotics treated seeds are experimentally deprived of gut bacteria but their aposymbiotic state was confirmed by culture dependent and molecular techniques as described below.

### Confirmation of aposymbiotic status of experimental beetles

The aposymbiotic status of the newly emerged beetles was checked using the culture dependent technique and the qPCR analyses targeting the 16S rRNA genes, respectively. Ten normal and ten antibiotics treated beetles, were randomly selected and individually dissected. Individual guts were removed and homogenized and the homogenate was serially diluted, spread on standard Luria-Bertani (LB) agar plates (10 g tryptone, 5 g yeast extract, and 10 g NaCl in 950 mL deionized water at pH 7.0) and incubated at 30 °C overnight. The Colony Forming Units (CFU) resulting from the bacterial colonies were calculated in each group. The estimation of cultivable bacteria in symbiotic beetles (normal) gave 6.514 × 10^5^ ± 13.72 × 10^2^ CFUs g^−1^.gut^−1^ while the antibiotic treated beetles gave 254 ± 9.32 CFUs g^−1^.gut^−1^ representing just 0.039% of the total bacterial communities of the normal beetles (Independent T-Test, F = 19.65; t = 11.502; df = 1, 28; P < 0.0001).

The bacterial genomic DNA was extracted from ten individual gut suspensions (earlier prepared) using the CTAB/SDS method^57^ and the recovered DNA samples were checked for quality by spectrophotometry and purified. Five nanograms of purified DNA was used per qPCR reaction targeting the bacterial 16S rRNA gene using universal primers 27 F/533R. Each sample was prepared by pooling 10 individual guts representing 10 replicates from symbiotic and aposymbiotic beetles each, using SYBRGreen in 384-well plates on an ABI 7300 Real-Time PCR detection system (Applied Biosystems Inc, Foster City, CA, USA). The 18 S rRNA was used as internal control gene for the determination of gut bacterial content from each sample. The gut microbial communities from the symbiotic beetles gave 10.01 × 10^6^ ± 2.4 × 10^6^ CFUs per gut while in aposymbiotic ones, an average gut bacterial content of 2.5 × 10^6^ ± 6.08 × 10^4^ CFUs (Independent T-Test, F = 89.692; t = 1.835; df = 1, 18; P < 0.0001). After this estimation, the antibiotic treated beetles were confirmed to be aposymbiotic (which is a conventional relative term, used widely in similar experimental settings, see refs^2^^,^^3^^,^^4^^,^^38^) and were used in subsequent experiment.

### Bioassays

Symbiotic and aposymbiotc beetles were assayed separately. The test was carried out in Petri dishes (Ø = 90 mm), each containing 10 g of artificial cowpea seeds divided into three treatment groups (EO, DDVP & Control) of 15 replicates each. Each treatment group comprised three biological replicates and each biological replicate contained 5 individual beetles. The EO groups (seeds treated with 10 µL of EO per gram of overall seed weight), the DDVP groups (seeds treated with 15 µL of DDVP per gram of overall seed weight), and finally the control groups (seeds treated with DMSO only). The working concentrations of EO and DDVP represent their effective concentration (EC_50_) determined after a dose-response assessment among several dosages (Fig. S2, Supplementary 1).

Five *C*. *maculatus* couples were introduced into each petri dish. The insects remained in contact with the grains for 6 days and adult mortality was recorded daily for 6 days until no mortality was seen^20^. The dead insects were removed and the surviving ones were left in respective experimental Petri dishes and selected for breeding of the next generation^58^. Emerging adults from different treatments (including controls) were used to set up separate lines. The resistance levels of experimental beetles were based on adult mortality rates at each generation and insects were considered dead if they were unable to turn over after gentle prod on their abdomen^26^. All treatments were maintained at 27 ± 3 °C temperature, 55 ± 5% relative humidity (RH) and 14:10 (Light: Dark) photoperiod. Only females were chosen for microbial analyses due to their high capacity to tolerate pesticides, shown by lower mortality rates recorded across generations compared to males (Fig. S3, Supplementary 1).

### Microbiological bioassay

Five surviving females from each of the three biological replicates of different treatment groups (Control, EO, and DDVP) at F_1_ and F_5_ generations, symbiotic and aposymbiotic were dissected and their individual guts were considered for gut microbial analysis based on high-throughput 454 pyrosequencing of V2-V3 regions of the 16S rRNA gene^59,60^. The three sequencing samples were randomly selected among replicates which show higher tolerance to each pesticide. To evaluate the effects of long term exposure to DDVP and EO on gut microbiota, gut samples from all treatments at F_1_ and F_5_ generations were considered for the analysis.

### Insect dissection

Five insects from each of the three biological replicates (making 15 individuals per treatment group) were collected, anesthetized by placing them at −20 °C for 30 minutes. Soon after, they were washed in 70% ethanol for 2 min and rinsed 3 times in sterile distilled water before dissection. To prevent contamination of the samples, the dissection was carried out aseptically with two pairs of sterilized forceps on a sterilized glass slide spread with 50 µL of sterile distilled water under a stereomicroscope^50^. The whole process was done in a laminar flow hood to avoid aerial contaminations. The intact guts were individually and separately transferred into Eppendorf tubes containing 750 μL TE buffer (10 mM Tris-Cl, pH 8; 1 mM EDTA, pH 8), disaggregated and manually homogenized with an Eppendorf adapted pestle and directly inoculated onto LB agar plates^61^. The homogenate was used for bacterial colony screening and total DNA extraction.

### DNA Extraction, molecular cloning and 454-Pyrosequencing

Genomic DNA samples were extracted using the CTAB method^57^ on five individual gut homogenates from each biological replicate and treatment. The quantity and quality of extracted gDNA were determined using a NanoDrop Spectrophotometer (Eppendorf AG, Germany) and stored at −20 °C prior to the analysis. The pyrosequencing of V2-V3 hyper-variable regions of the 16S rRNA gene amplicons was carried out on a 454 GS FLX Titanium system (Roche, Penzberg, Germany)^60,62^. Universal barcoded primers Ba27F (5′-AGA GTT TGA TCM TGG CTC AG-3′) and Ba519R (5′-TAT TAC CGC GGC KGC TG-3′) containing A and B sequencing adaptors (454 Life Sciences), key sequences and multiplex identifiers were used to prepare the pyrosequencing amplicons^63^. Before pyrosequencing, amplicons were purified from gel using AxyPrep DNA gel extraction kit (Axygen Scientific, Inc.), quantified using Quant-iT Pico Green dsDNA assay (Invitrogen, Germany) and pooled in an equimolar ratio before being subjected to emulsion PCR and breaking. GS FLX Titanium chemistry was used to perform pyrosequencing from A-end according to the supplier protocol.

### Statistical analysis

All mortality data from each treatment group were corrected using Abbott’s formula^64^ and tested for homogeneity of variances using Levene’s tests. To check important factors that influence the susceptibility of the experimental beetles, variables of overall mortality were analyzed (each one separately) using the ordinary least squares regression model with symbiotic status, pesticides and generations as effects. Multivariate tests (using DDVP, EO and DMSO data as dependent variables) and multifactorial analyses (using the symbiotic status and experimental generations as fixed factors) were performed to analyze the variations of mortality within and between all the parameters (symbiotic status, experimental generations and pesticide treatments). The one-way analysis of variance (ANOVA) was used to analyze differences in mortality and sequencing data (Table 1). Colony Forming Units of antibiotics treated and untreated samples were analyzed using an independent T-Test. Tukey’s HSD test at P = 0.05 significance, was used for mean separations within and between treatments. Colony Forming Units (CFU) data were log-transformed to narrow down data variations. Statistical analyses were carried out using SPSS 20.0 software (Statsoft Inc, Carey, J, USA). Bray–Curtis and unweighted UniFrac distance matrices were used to calculate the beta diversity and the analysis of similarity (ANOSIM) was performed to evaluate the similarity of *C*. *maculatus* gut bacterial community among treatments using the software PRIMER 7.0. All results (alpha and beta diversity, survival, & sequence data) are presented as mean ± standard deviations (SD) of the three biological replicates. OriginPro 8.5.1 software was used to construct graphs.

### Bioinformatics analysis

Bioinformatics analysis was done separately for each of the three samples (control, DDVP and EO) using Quantitative Insights Into Microbial Ecology (QIIME) 1.9.0 pipeline^65^. Raw pyrosequencing reads (54,885) were denoised using the “pre.cluster” command (http://www.mothur.org/wiki/Pre.cluster) in mothur platform (version 1.25.0; http://www.mothur.org)^66^. Any mismatch in the barcode, more than two primer mismatches, homopolymers of more than 8 bases and sequences less than 160 bp were discarded for the analysis. Chimeric sequences were identified and removed using UCHIME, and the remaining unique sequences were clustered into Operational Taxonomic Units (OTUs) using the Greengenes database filtered at 97% similarity, which is the consensus threshold for species boundaries^67^. A total of 47,766 high-quality pyrosequencing reads were produced according to barcode- and primer-sequence filtering, and were used for further analysis.

To obtain additional information regarding species diversity composition, sequence reads were subjected to redundancy treatment with Mothur software to count the number of identical tags. Taxonomic classification of OTUs was done via the ribosomal database project (RDP), with naïve Bayesian settings at 0.5 confidence threshold^68^. Rarefaction analysis was used to compare sequencing depth and bacterial species richness within and between F_1_ and F_5_ generations. Only OTUs that reached 97% identity level were used for alpha diversity (Shannon and Simpson) and richness (Chao1) analysis. Dataset was normalized at 300 sequences per sample, and this rarified threshold was used to identify the OTUs which were highly affected by the pesticides^69^. Mothur program was used to construct Venn diagram to show OTUs that are unique or shared between treatments (Control, DDVP and EOs)^70^. Principal components analyses were generated using Bayesian algorithms^71^ and dendrograms showing the similarity of bacterial communities in different treatments were constructed through a *jackknife*-beta-diversity script from QIIME^65^.

### Ethical Statement

*Callosobruchus maculatus* is indigenous in Cameroon. *Lippia adoensis* fresh leaves were collected from the experimental field of the Department of Biological Sciences, Faculty of Science, University of Ngaoundere (Cameroon) and cowpea seeds were bought from local farmers. Therefore, no permit was required for their collection and manipulation. The study was approved by the scientific committee of the Faculty of Science and validated by the Scientific Committee of the University of Ngaoundere (Decision no. 2015/093) in a full PhD Program assigned to the author M.A.

## Supplementary information

Dataset 1

## Data Availability

Metagenomic data generated from this study has been submitted to the Sequence Read Archive (SRA) of NCBI under the BioProject ID: PRJNA530839. Other additional data are available from corresponding authors upon request.
