# Supplementary material for: Gut bacteria of the cowpea beetle mediate its resistance to dichlorvos and susceptibility to Lippia adoensis essential oil
Source: Sci Rep. 2019 Apr 23;9:6435. doi: 10.1038/s41598-019-42843-1 (PMC6478711; doi:10.1038/s41598-019-42843-1)
Supplement: Supplementary file 1 — Dataset 1 [file 41598_2019_42843_MOESM1_ESM.docx]

**Gut bacteria of the cowpea beetle mediate its resistance to dichlorvos and susceptibility to *Lippia adoensis* essential oil**

Mazarin Akami^1,3*^, Nicolas Yanou Njingtang^1^, Olajire A. Gbaye^2^, Awawing A. Andongma^3^, Muhammad Adnan Rashid^3^, Chang-Ying Niu^3^, and Elias Nchiwan Nukenine^1^

**Electronic supplementary materials 1 (ESM 1)**

1. Chemical characterization of *Lippia adoensis* essential oil

The chemical constituents (%) of the essential oil extracted from young leaves of *L. adoensis* harvested from Mbe, in the Adamawa region of Cameroon has been published and is freely available online[^1^](#_ENREF_1).

1. **Antibiotics selection**

Antibiotics Ciprofloxacin and Gentamycin were selected among three tested ones for their capacity to reduce to the minimum the gut bacterial communities from *C. maculatus*.





**Figure S1.** Variation of *Callosobruchus maculatus* gut associated bacteria based on Colony Forming Units (CFU) after antibiotics (Ciprofloxacin and Gentamycin) treatments. Means with different letter(s) are statistically different after comparison with Tukey's HSD Test at *p* = 0.05.

1. **Dose response assessment**

The aim of dose response evaluation was to find the effective concentration (EC_50_) of each pesticide to be used in susceptibility test. Five dosages (0, 5, 10, 20 and 40 µL) of EO and DDVP were dissolved in 1 mL of dimethylsulfoxide (DMSO) and were separately introduced in 250 mL glass jars, each containing 20 g of cowpea. The jars were hand shaken for 5 minutes and left open for about 20 minutes at room temperature to allow complete evaporation of the solvent[^1^](#_ENREF_1). Twenty beetles (10 males and 10 females) aged 1-2 days old were introduced into each jar and sealed with perforated lid covered with thin mesh. Cumulative adults’ mortality was recorded daily until no dead insects were seen (about one week post-exposure). Data generated from this trial served to determine the working concentrations of EO and DDVP based on their EC_50_ computably determined. These EC_50_ were used in subsequent experiments.





**Figure S2.** Dose-response of *Callosobruchus maculatus* to gradual concentrations of Dichlorvos and Essential Oil. Means with different letter(s) are statistically different after comparison with Duncan's Test at *p* = 0.05.

1. **Preparation of artificial cowpea seeds**
2. **Ingredients**

**Table S1:** Ingredients for experimental diets

| **No.** | **Ingredients (g)** | **Control** | **EO** | **DDVP** | **Antibiotics** |
| --- | --- | --- | --- | --- | --- |
| 1 | Cowpea flour | 100 | 100 | 100 | 100 |
| 2 | Methyl-p-hydroxybenzoate | 0.8 | 0.8 | 0.8 | 0.8 |
| 3 | Cholesterol | 0.5 | 0.5 | 0.5 | 0.5 |
| 4 | Choline chlorite | 0.36 | 0.36 | 0.36 | 0.36 |
| 5 | L-Ascorbic acid | 1.1 | 1.1 | 1.1 | 1.1 |
| 6 | Sodium benzoate | 0.4 | 0.4 | 0.4 | 0.4 |
| 8 | Gelatin | 8 | 8 | 8 | 8 |
| 9 | Gentamycin (4µg/ml) | - | - | - | 800 |
| 10 | Ciprofloxacin (10µg/ml) | - | - | - | 1,000 |
| 11 | EO (0. 17 mL/g) | - | 17 | - | - |
| 12 | DDVP (0.2 mL/g) | - | - | 20 | - |
| 13 | DDW (mL) | 80 | 80 | 80 | 80 |

DDW: Deionized Distilled Water; DDVP: *O,O*-dimethyl-*O*-2,2-dichlorovinylphosphate; EO: essential oil. The ingredients are necessary to make 100 gram of artificial seeds. For higher quantity, please adjust the amounts accordingly.

Symbiotic beetles were produced from normal artificial seeds containing no antibiotics and pesticides. Mineral elements removed during the seed decortication were added in order to boost the females’ fecundity (supplementary).

Normal females were allowed to lay eggs on antibiotics treated diets to produce aposymbiotic beetles. To check the aposymbiotic status of the newly emerged beetles, ten normal and ten antibiotics treated beetles were randomly selected and individually dissected. Individual guts were homogenized, serially diluted, spread on standard LB Agar plates and incubated at 30ºC overnight. The aposymbiotic status was confirmed after observing significant difference of colony forming units (CFU) between the antibiotic treated beetles and the normal ones.

1. **Preparation procedures of artificial seeds**

Ingredients number 2-8 were mixed in DDW with a magnetic agitator for 20 minutes. The cowpea flour was then added progressively while swirling until obtaining a homogenous paste. The paste was divided into four parts representing the four treatment groups, before adding EO or DDVP or antibiotics earlier dissolved in DMSO. In each treatment group, the artificial seeds were allowed to lyophilize for 24h before removing them from the mold, and equilibrated with atmospheric moisture, prior to coating with 10% gelatin[^2^](#_ENREF_2). All the procedures were carried out under aseptic conditions.

1. **Susceptibility based on sex of symbiotic and aposymbiotic beetles**

The mortality of males and females were separately evaluated to see their differential susceptibility to both pesticides across experimental generations. These were important as it determined the most tolerant beetle on which the microbial analysis was carried out.

As shown in Figure S3 below, the susceptibility of experimental beetles was significantly affected by sex. Females beetles showed significantly lower mortality rates to both pesticides than males across generations (Ordinary Least Squares Regression Model, F = 211.59; df = 3; r^2^ = 0.98; t = 4.6593; P ˂ 0.001). In general, higher adult’s mortality was recorded in DDVP treatments. No mortality was recorded in control groups.


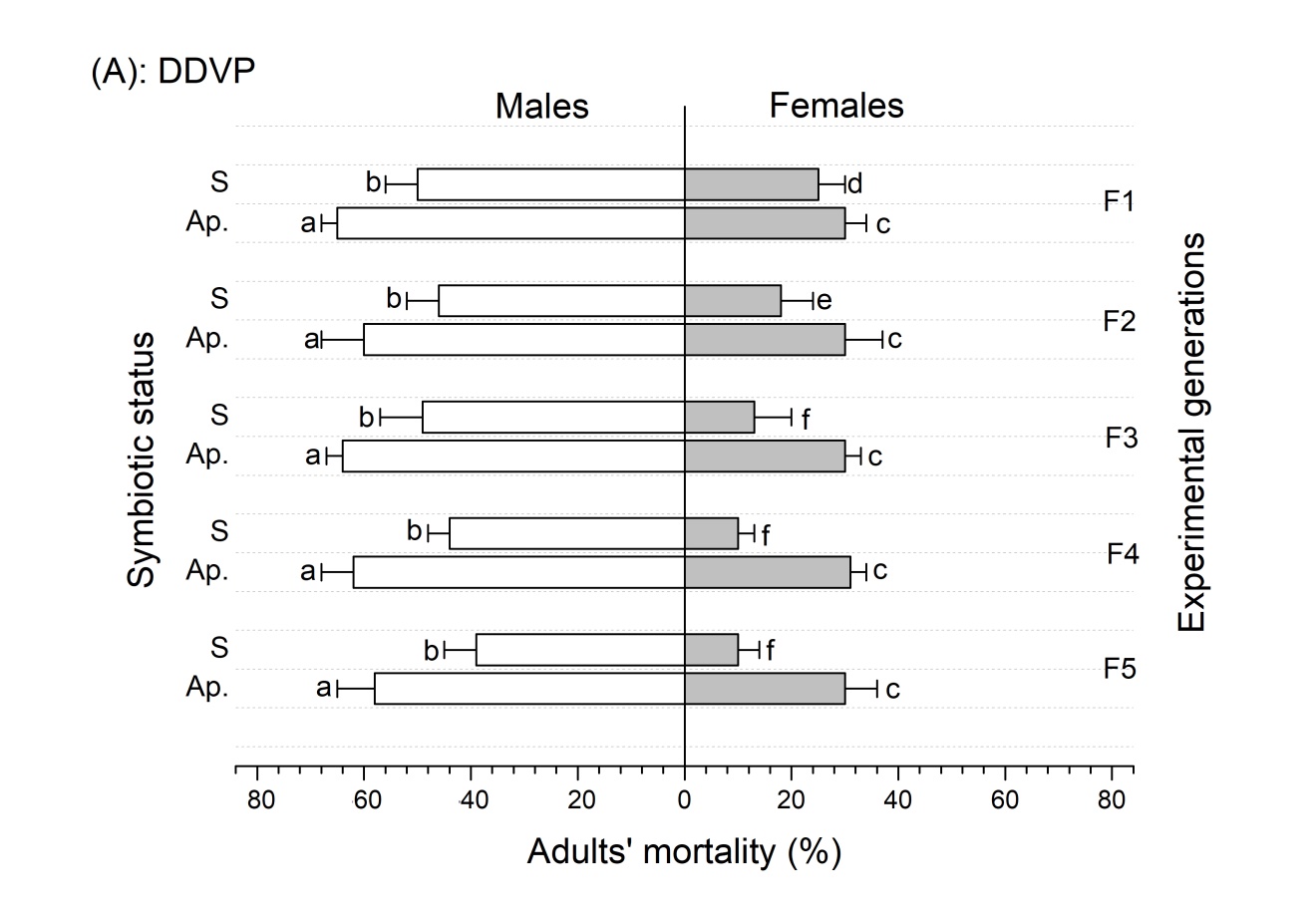


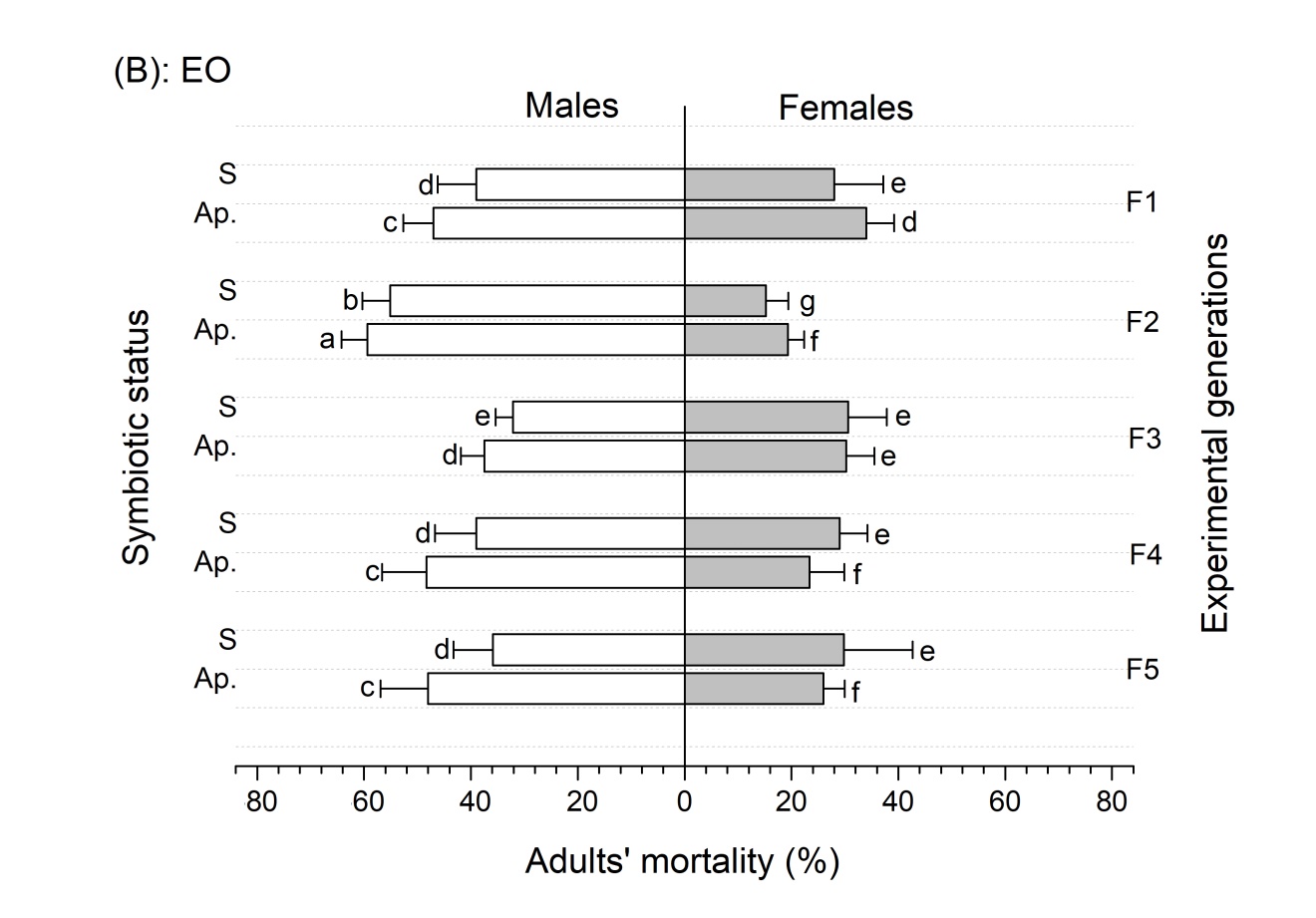


Figure S3. Variation of the susceptibility of symbiotic and aposymbiotic cowpea beetles over five generations. Means with different letters between generations of each pesticide treatment are significantly different after comparison with parametric New Duncan's Multiple Range Test at *p* = 0.05.

**References**

1 Mazarin, A., Nukenine, E. N., Niu, C. & Vincent, F. V. Synergistic effects of wood ash and essential oil on fecundity, pupal eclosion and adult mortality of C*allosobruchus maculatus* (Coleoptera: Bruchidae) cowpea seed weevil. *American Journal of Experimental Agriculture* 11, doi:10.9734/AJEA/2016/25306 (2016).

2 Guo, F. *et al.* Antagonistic regulation, yet synergistic defense: effect of bergapten and protease inhibitor on development of cowpea bruchid *Callosobruchus maculatus*. *PloS one* 7, e41877, doi:10.1371/journal.pone.0041877 (2012).
